# Investigation into safflower injection as a prophylactic treatment for retinal vein occlusion in a rabbit model

**DOI:** 10.1038/s41598-024-58734-z

**Published:** 2024-04-05

**Authors:** Junling Li, Zhenfeng Guo, Jianguo Wu

**Affiliations:** Department of Ophthalmology, Tianjin Beichen Hospital, Affiliated Hospital of Nankai University, No. 7 Beiyi Road, Beichen District, Tianjin, 300400 China

**Keywords:** Drug discovery, Medical research

## Abstract

The study aimed to assess the effect and mechanism of safflower injection in preventing retinal vein thrombosis in rabbits. Twenty healthy adult pigmented rabbits were randomly assigned to either the experimental group, receiving safflower injection, or the control group, receiving normal saline. After two weeks of treatment, blood samples were collected to analyze platelet adhesion and aggregation rates. Photodynamic therapy was applied to induce occlusion in the target retinal vein. Fundus photography and fluorescein angiography were recorded using a dynamic microscopic monitoring system, and laser speckle imaging was employed to assess blood flow in the affected vein. The experimental group exhibited significantly lower rates of platelet adhesion and aggregation compared to the control group. Following the induction of retinal vein occlusion, the experimental group showed a lower complete occlusion rate of the target retinal vein. Although initial blood flow in the target vein was similar between groups, the blood flow at 1, 3, and 5 min post-occlusion was significantly higher in the experimental group. Safflower injection delayed retinal vein thrombosis formation, preserved blood flow in the affected retinal area, and reduced platelet adhesion and aggregation. These effects facilitated vascular reperfusion within a limited timeframe.

## Introduction

Retinal vein occlusion (RVO) is a significant chronic retinal vascular disorder and the second most common cause of vision impairment after diabetic retinopathy. A multicenter study demonstrated that, within three years of an RVO event, visual acuity declined by 40% in 40% of patients, with 12% experiencing a decrease to less than 0.1^1^. Current treatments, including fundus laser photocoagulation, corticosteroid therapy, and anti-vascular endothelial growth factor (anti-VEGF) injections, aim to mitigate vision loss primarily due to macular edema and neovascularization. While these approaches offer some therapeutic benefits, they fall short of being completely safe or ideal, with no established method to directly enhance perfusion^2,3^. RVO is commonly associated with multifactorial damage to the vascular endothelium, leading to thrombosis^4,5^. Research indicates that vascular injury triggers platelet activation through intricate receptor-ligand interactions, culminating in thrombosis and impaired blood flow^6–8^, which can result in a spectrum of severe complications^9,10^. Evidence suggests that inhibiting platelet activation or aggregation could prevent thrombosis^11,12^, potentially reducing the damage caused by RVO.

Safflower injection, primarily composed of safflower yellow (SY), carthamone, carthamidin, and neocarthamin, has been identified by modern pharmacology as an agent that can prevent thrombosis. SY, the main active component, inhibits thrombosis by blocking adenosine 5'-diphosphate (ADP) receptors, reducing thromboxane A2 synthesis, enhancing prostacyclin production, and diminishing the activity of plasma plasminogen activator inhibitors^13^. Despite the widespread clinical application of SY and its reported diverse pharmacological effects, research on its role in preventing retinal vein thrombosis is scarce. This study employs a rabbit branch retinal vein occlusion (BRVO) model, induced via photodynamic therapy (PT)^14^, along with a dynamic microscopic monitoring system^15^ and laser speckle imaging (LSI)^16^ techniques. These tools allow for the real-time quantitative assessment of retinal vein blood flow, facilitating the investigation of the safflower injection's efficacy and mechanism in thwarting RVO-induced thrombosis in rabbits.

## Material and methods

### Experimental animals and grouping

*Animal selection*: We utilized twenty healthy adult pigmented rabbits, each weighing between 2 and 3 kg. The rabbits were acquired from Beijing Vital River Laboratory Animal Technology Co., Ltd. and were housed under controlled environmental conditions with a 12-h light/dark cycle, provided with ad libitum access to food and water.

*Ethical approval*: The Institutional Animal Care and Use Committee of Tianjin Medical University approved all animal experiments. This study adheres to the Animal Research: Reporting of In Vivo Experiments (ARRIVE) guidelines. All methods were conducted in strict accordance with the relevant guidelines and regulations.

*Grouping procedure*: The rabbits were randomly divided into two groups, comprising ten animals each. The randomization was conducted using a random number table method to ensure unbiased allocation. The two groups included:

*Experimental group*: Rabbits in this group received safflower injection treatment.

*Control group*: Rabbits in this group were treated with an equivalent volume of normal saline as a placebo.

*Treatment administration*: The experimental group received intramuscular injections of safflower injection (0.3 ml/kg, Ya’an Sanjiu Pharmaceutical Co., Ltd.) daily for a continuous period of two weeks. The control group received intramuscular injections of normal saline, following the same dosage and schedule.

*Identification*: Each rabbit was individually marked for identification to ensure accurate and consistent treatment and data collection throughout the study.

## Measurement of platelet adhesion rate and aggregation rate

*Blood collection and anesthesia*: 30 Minutes following the final treatment administration, rabbits from both the experimental and control groups were anesthetized using an intramuscular injection of sumianxin (Jilin Province Dunhua Shengda animal drug Co., LTD) at a dose of 2 ml/kg. Subsequently, 4 mL of blood was drawn from the ear vein of each rabbit for further analysis.

*Platelet adhesion rate measurement*: From the blood sample collected, 1.5 mL was utilized to calculate the platelet adhesion rate. The assessment was performed using an in vitro thrombosis induction–platelet adhesion instrument (Model GX-964), employing the glass vial method.

*Platelet aggregation rate measurement*: The remaining 2.5 mL of the blood sample was transferred into a test tube containing 0.3 mL of 3.8% sodium citrate to prevent coagulation. The mixture was gently mixed to ensure proper anticoagulation and then centrifuged to separate the platelets from other blood components. The platelet-rich plasma obtained was used to measure the rate of platelet aggregation. The aggregation was induced by the addition of ADP supplied by Boster Biological Technology Co., Ltd., Wuhan, China. The rate of platelet aggregation within a 5-min period was measured and recorded using an automatic platelet aggregometer (Model PRECIL LBY-NJ4A).

## PT-induced BRVO model

After the process of blood collection, the next step was to establish a model of BRVO using a method referenced from the literature^14,15^. The procedure for creating this model is as follows:*Rose bengal injection*: The first step in the procedure involved the administration of Rose Bengal (Sigma, St. Louis, MO), a photosensitizing agent, at a dosage of 10 mg/kg. This compound was injected into the ear vein of the rabbit. Rose Bengal is known to localize in the vascular endothelium and, when activated by light, can produce reactive oxygen species that lead to vessel occlusion.

*Correction of anisometropia*: Before inducing BRVO, any anisometropia in the experimental eye was corrected by corneal contact lens (CCL). This step is crucial to ensure that the laser targets the intended area accurately and does not affect the surrounding healthy tissue.

*Target retinal vein*: The selected target for inducing BRVO was the trunk of the retinal vein situated at a distance of 0.5–1.0 mm from the optic disc. This specific location is chosen because it is a common site where natural BRVO occurs in humans.

*Laser irradiation*: After 1 min, Rose Bengal circulated through the bloodstream and reached the target retinal vein, the vein was then exposed to continuous irradiation using a 550-nm laser. The laser parameters were set to a power of 100 mW and a spot size of 100 µm in diameter. The irradiation lasted for 5 min. The interaction between the laser light and Rose Bengal produces a photodynamic reaction, leading to the occlusion of the targeted retinal vein and the creation of a BRVO model.

## LSI to monitor blood flow in the target vein region of the fundus

The LSI technique was employed to monitor retinal blood flow in the target vein region of the fundus following the occlusion induced by PT. The procedure is detailed below:

*LSI equipment and parameters*: A laser speckle imager (MoorFLPI2, Moor, UK) was used to detect the retinal blood flow. The device operates at a wavelength of 785 nm with a power output of 50 mW, providing continuous radiation. The imaging process was guided by the MoorFLPI-2 Measurement V1.0 software, which set the parameters for the image acquisition:

- CCD (Charge Coupled Device) Resolution: 576 × 768 pixels
- Temporal Filtering Rate: 25 frames per second
- Acquisition Frequency: 5 Hz
- Exposure Time: 0.5 s
- Continuous Acquisition Time: 5 min

*Image acquisition and analysis*: The fundus vessels were visualized clearly on the screen using the image acquisition device. The diameter of the target vein and the corresponding area of 0.2 mm^2^ were marked. The built-in analysis function of the program was utilized to measure and record the blood flow in the target retinal vein area at different time points (0 min, 1 min, 3 min, and 5 min).

## Fundus photography and fluorescein angiography

The assessment of the retinal vein occlusion model and the circulatory status of the target vein in the rabbits was performed using fundus photography and FFA. These imaging techniques were applied at two critical time points: before the induction of the model and after the construction of the RVO model. An APS digital fundus angiography system from Chongqing Kompass Technology Co., Ltd. was employed for both fundus photography and FFA.

*Criterion for complete occlusion*: The specific criterion used to define complete occlusion of the target retinal vein was the absence of fluorescein filling within the target vein for a duration of 45 s after the construction of the model. If FFA showed no filling with the fluorescent dye in the target vein during this time frame, it was considered a complete occlusion.

### Data analysis and statistics

**Figure 1:**
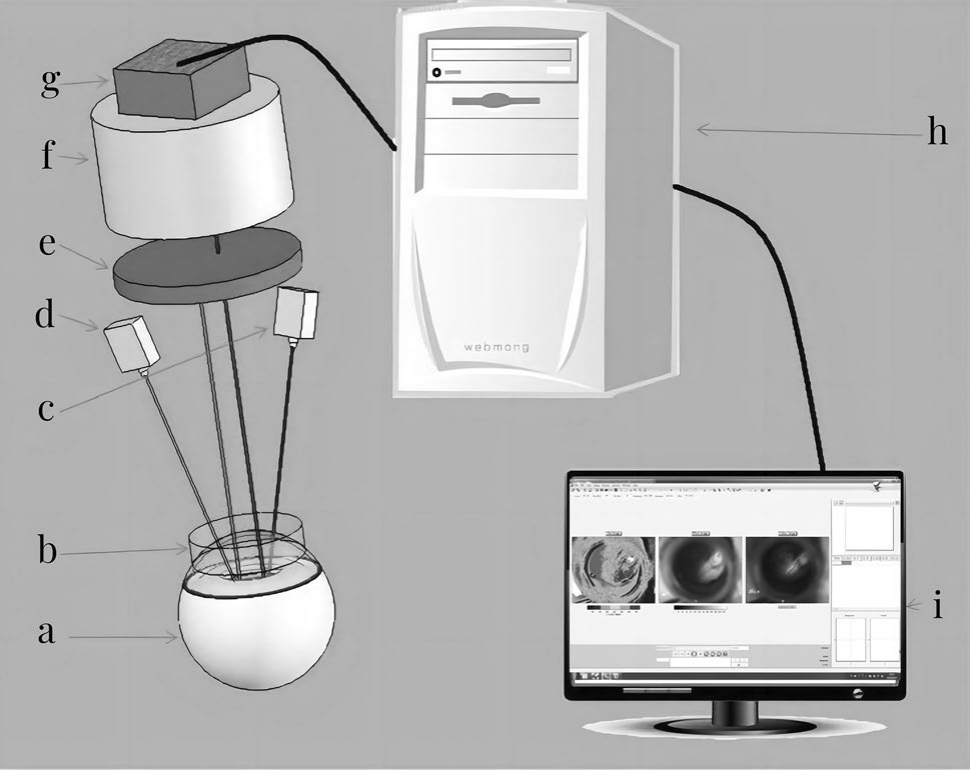
Schematic Diagram Description: The schematic provided illustrates the setup used for synchronous LSI detection of retinal blood flow following photodynamic induction of thrombosis. The key components of this setup include: **a** Rabbit Eyeball: The subject of the experiment. **b** CCL: Placed on the rabbit's cornea to facilitate imaging and focusing. **c** 785-nm Laser Emitter: Used for the LSI to detect blood flow. **d** 532-nm Laser Emitter: Used for the PT to induce thrombosis. **e** Optical Filter: To filter out unnecessary wavelengths and enhance image quality. **f** Microscope: For magnifying the retinal area being imaged. **g** High-Resolution CCD: The imaging sensor capturing the speckle pattern. **h** Processor: The component processing the data from the CCD. **i** Liquid Crystal Display (LCD): To display the images and data for analysis.

SPSS 22.0 software was utilized for the statistical analysis. Mann–Whitney U text was employed to compare the platelet adhesion rates, aggregation rates, and blood flow rates in the target retinal vein areas at various time points post-model induction between the two groups. The chi-square test was used to compare the rate of complete RVO between the two groups. A P-value of less than 0.05 was considered statistically significant (Fig. 1).

## Results

### Effect of safflower injection on the platelet adhesion rate and aggregation rate in rabbits

The experimental group showed a significantly lower platelet adhesion rate compared to the control group, and the 1-min platelet aggregation rate (PAG1) and 5-min platelet aggregation rate (PAG5) were significantly lower in the experimental group compared to the control group (*P* < 0.05) (Table 1).

**Table 1:** Comparison of platelet aggregation rate and aggregation rate between the two groups.

	Median(P25, P75)		Mann Whitney test statistic *U* value	Mann Whitney test statistic *z* value	*p*
	Control group (*n* = 10)	Experimental group (*n* = 10)			
Platelet adhesion rate (%)	35.060(26.5,37.4)	28.195(21.6,33.5)	22.000	-2.117	0.034*
PAG1	45.650(40.9,47.8)	33.170(26.9,36.3)	4.000	-3.477	0.001**
PAG5	41.320(38.8,48.6)	30.980(26.5,32.4)	5.000	-3.403	0.001**

* *p* < 0.05 ** *p* < 0.01.

## Effect of safflower injection on photodynamically induced BRVO in rabbits

### Comparison of fundus photography and FFA results between the two groups

**Figure 2:**
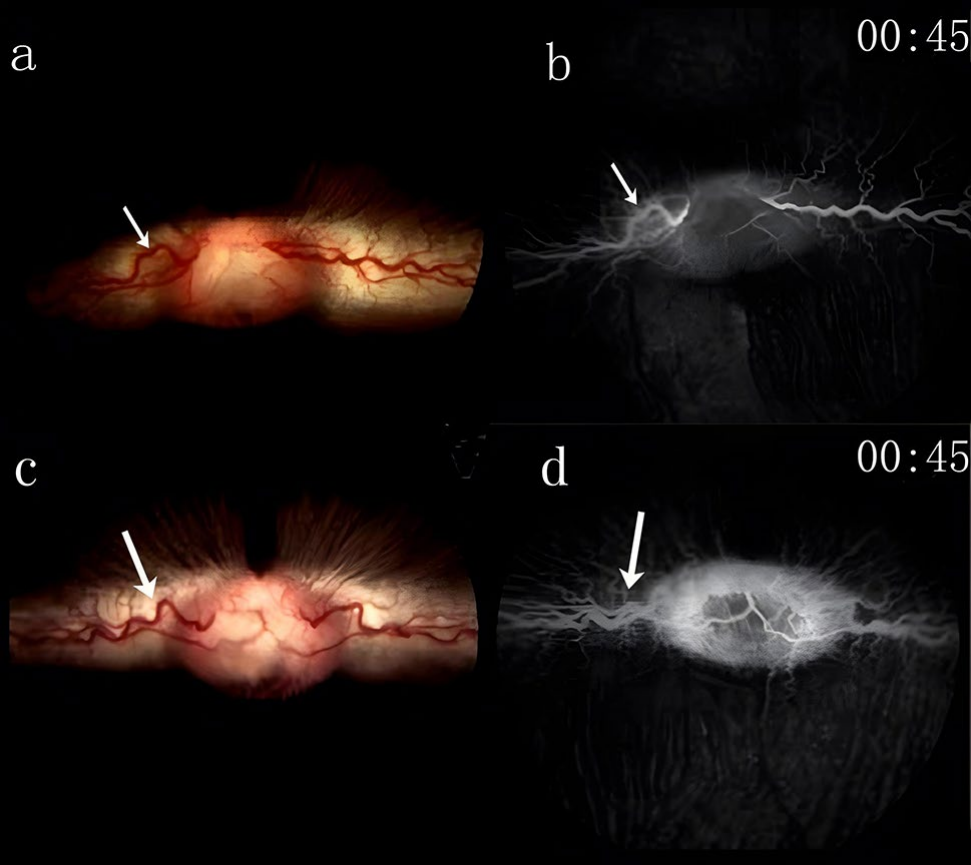
Fundus photographs and FFA images captured the condition of rabbit eyes in both groups prior to the experiment, with arrows indicating the target veins. **a** and **b** confirm that, in the experimental group, there was no obstruction and blood flow within the target vein was normal before the application of PT to induce thrombosis. Similarly, **c** and **d** illustrate that, in the control group, the target vein was also free from any blockage, allowing for unimpeded blood flow before the initiation of thrombosis through photodynamic treatment.

*Baseline observations*: At the time of enrollment, the vitreous and retinal structures of the rabbits were clearly visible in both groups. Fundus photography (referred to as Fig. 2a,b for the experimental group and Fig. 2c,d for the control group) did not reveal any abnormalities such as hemorrhage, edema, or obstruction in either group.

**Figure 3:**
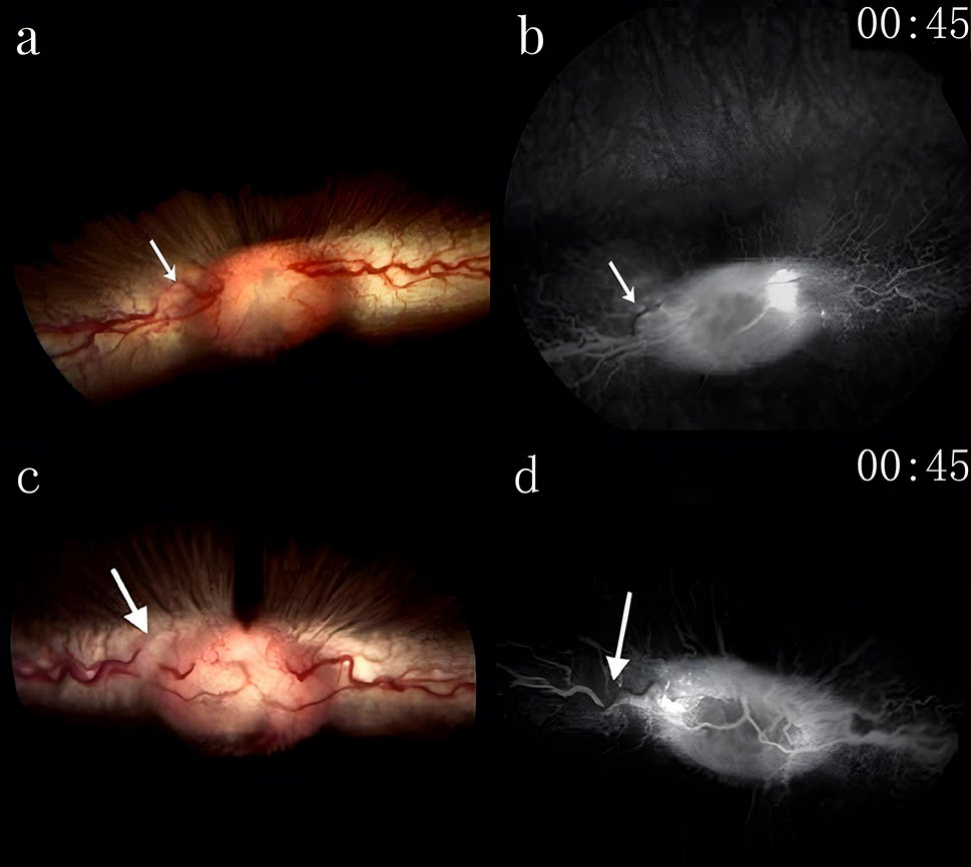
After the experiment, fundus photographs and FFA images revealed the outcomes of photodynamically induced thrombosis in the rabbit eyes of both groups, with arrows highlighting the target veins. **a** and **b** depict partial occlusion of the target vein in the experimental group, indicating that the thrombosis did not result in a complete blockage. Conversely, **c** and **d** show that in the control group, the target vein experienced complete occlusion, demonstrating a total blockage as a result of the photodynamic thrombosis.

*Post-thrombosis observations*: After PT was used to induce thrombosis in the retinal veins, changes were observed in the laser-irradiated area. Fundus photography showed retinal edema and incomplete occlusion of the target vein in the experimental group (Fig. 3a,b), and complete occlusion in the control group (Fig. 3c,d).

*Incidence of complete occlusion*: In the experimental group, complete occlusion of the target vein was observed in 4 out of 10 rabbit eyes (40%). In contrast, the control group exhibited a 100% incidence rate of complete occlusion, with all 10 rabbits affected.It is a significant difference in the occlusion rate between the two groups (*P* < 0.01) (Table 2).

**Table 2:** Comparision of the occlusion rate on RVO between the two groups.

Group	Occlusion rate (%)		Total	χ2	*p*
	Incomplete	Complete			
Experimental group (n = 10)	6(100.00)	4(28.57)	10(50.00)	7.200	0.007**
Control group (n = 10)	0(0.00)	10(71.43)	10(50.00)		
Total	6	14	20		

* *p* < 0.05 ** *p* < 0.01.

## Comparison of retinal blood flow in the target vein region between the two groups

Table 3 presents data on the blood flow within the target vein region of both the experimental and control groups at various time intervals. Initially, at the 0-min mark, there was no statistically significant difference in blood flow between the two groups, with a P-value greater than 0.05. However, as time progressed, the experimental group showed significantly higher blood flow compared to the control group at the 1-min, 3-min, and 5-min time points, with the differences being statistically significant (*P* < 0.01).

**Table 3:** Comparison of blood flow in the target retinal vein region between the two groups at different time points.

	Median(P25, P75)		Mann Whitney test statistic *U* value	Mann Whitney test statistic *z* value	*p*
	Control group(*n* = 10)	Experimental group(*n* = 10)			
0 min (PU)	1733.105(1629.8,1754.1)	1737.685(1714.5,1757.4)	43.000	− 0.529	0.597
1 min (PU)	1056.265(942.0,1162.1)	1324.250(1221.7,1398.8)	8.000	− 3.175	0.001**
3 min (PU)	220.750(207.2,258.5)	751.050(331.8,792.9)	10.000	− 3.024	0.002**
5 min (PU)	41.655(32.4,48.6)	244.560(74.0,389.9)	1.000	− 3.704	0.000**

* *p* < 0.05 ** *p* < 0.01.

## Discussion

RVO is a common retinal vascular condition that currently lacks treatment options that are both safe and fully effective^1–3^. From the standpoint of comparative medicine, it is essential to validate the prophylactic effects of potential treatments for RVO using an appropriate animal model^15,17^. This validation necessitates the acquisition of trustworthy and quantifiable experimental blood flow data in real time, which can be achieved through the use of a dynamic microscopic monitoring system combined with LSI.

In alignment with a "prevention first" approach, the current study employed a safflower solution^18^—a traditional Chinese medicinal compound—and preadministered it to rabbits. The aim was to investigate whether the safflower injection could enhance platelet adhesion and aggregation, preserve local blood flow in a rabbit model of RVO, and thereby potentially extend the window of opportunity for vascular reperfusion. The ultimate goal was to assess whether this treatment could reduce the rate of complete occlusion in the target veins, offering a new avenue for RVO management.

Platelets are fundamental in the development of venous thrombosis^19,20^. They are involved in the formation of blood clots, which can lead to conditions such as RVO . SY, the primary active ingredient in the safflower injection solution, has been shown to impact the expression of activated glycoproteins on the membranes of platelets. It achieves this by affecting the activation of downstream signaling pathways of ADP receptors, which play a crucial role in platelet aggregation. ADP-induced platelet aggregation is a process where ADP binds to its receptors on platelets, triggering a series of intracellular events that result in platelet activation and clumping. By inhibiting this process, SY can effectively reduce the likelihood of platelet aggregation.

The current study demonstrated that pre-administration of safflower injection in a rabbit model led to a decrease in both the platelet adhesion rate and the platelet aggregation rate within the target veins. These effects are significant as they contribute to delaying the onset of thrombosis, which is the pathological formation of blood clots within a blood vessel. By interfering with platelet adhesion and aggregation, safflower injection may help maintain blood flow and prevent complete occlusion of the veins, which is a critical outcome in the context of RVO prevention and treatment.

In thrombosis research, particularly in animal models, monitoring blood flow is a critical method for both verifying the successful establishment of the model and evaluating the efficacy of potential therapeutic agents^21^. LSI is considered one of the most reliable techniques for measuring blood flow^16^.

In this study, LSI was used to compare the blood flow rates in two groups of rabbits—those treated with safflower injection and a control group—immediately after the establishment of the thrombosis model (at 0 min). The lack of significant difference in blood flow rates between the two groups at this initial time point suggested that safflower injection does not enhance venous blood flow under normal circulatory conditions.

Following the construction of the thrombosis model, the target vein underwent vasodilation, but this was accompanied by a reduction in blood flow, which in some cases could lead to stagnation. Over time, blood flow decreased in both groups, which is expected as the model simulates the progression of thrombosis. However, the experimental group that received safflower injection maintained a significantly higher blood flow compared to the control group.

The study's findings indicate that safflower injection contributes to preserving blood flow in the vessels of the affected area through mechanisms that include, but are not limited to, reducing the rate of platelet adhesion and aggregation. By doing so, it delays the formation of thrombosis in the target vein and creates a therapeutic window during which vascular recanalization can occur. This suggests that safflower injection may have a protective effect in preventing venous thrombosis, such as RVO, and could be a beneficial intervention for maintaining blood flow and preventing complete vascular occlusion.

The insights from experimental and clinical studies of venous thrombosis^22^, underscore the time-dependent nature of the clinical outcomes associated with thrombotic events. The timing of interventions aimed at restoring blood flow is critical, with early restoration being particularly beneficial. Quick intervention can limit the size of the thrombus, reduce the extent of venous wall fibrosis, and minimize the long-term complications that often follow thrombotic events.

The study discussed here provides experimental evidence that administration of safflower injection prior to the induction of a thrombotic model in rabbits is effective in maintaining blood flow in the target retinal vein. By preserving blood flow, the safflower injection reduces the thrombus burden, which in turn results in a lower rate of incomplete occlusion in the target vein compared to the control group. These findings suggest that the safflower injection has a protective effect against the progression of thrombosis by improving hemodynamics, which is the flow and movement of blood.

The implication of these findings for clinical practice is significant. If safflower injection can delay the onset or progression of thrombosis in the clinical setting, it could extend the time window during which reperfusion-restoration of blood flow-can be successfully achieved. By doing so, it may reduce the severity of RVO and improve the overall clinical outcomes for patients.

This study suggests that safflower injection could be a promising therapeutic intervention for RVO, warranting further clinical investigation to validate its efficacy and safety in human subjects. Meanwhile, the study also recognizes the limitations inherent in using animal models to simulate human conditions. While the thrombi induced in the rabbit retinal vein through PT are similar to those found in human RVO, there are unavoidable differences between how diseases manifest in animal models and in human patients. Therefore, the results obtained from such studies must be approached with caution when considering their application to human medicine.

## Conclusions

The safflower injection, which is based on principles of traditional Chinese medicine, has been demonstrated to have a significant impact on the prevention of thrombosis by inhibiting platelet adhesion and aggregation. It helps to maintain blood flow in the vessels that could otherwise be compromised by RVO.

The integration of PT, dynamic microscopic monitoring, and LSI for model construction enables real-time and quantitative analysis of vascular microcirculation. This novel approach provides a robust method for assessing the efficacy of pharmacological interventions targeting vascular health.

To move forward, the research suggests that comprehensive clinical trials with a prospective design, involving multiple centers and a large sample of human participants, are necessary. These trials would aim to verify the effectiveness of safflower injection in reducing the incidence and severity of RVO and its associated complications in humans. If successful, these trials could pave the way for the integration of traditional Chinese medicine into mainstream therapeutic strategies for RVO, offering patients additional treatment options and potentially improving clinical outcomes.

## Data Availability

The datasets generated and analyzed during the current study are available from the corresponding author on reasonable request.
